# Application of Strain Elastography in Dentistry: A Systematic Review

**DOI:** 10.7759/cureus.70693

**Published:** 2024-10-02

**Authors:** Pragati Agarwal, Seema R Kambala, Surekha R Dubey, Anjali Bhoyar, Khushbu Doshi

**Affiliations:** 1 Department of Prosthodontics, Sharad Pawar Dental College and Hospital, Datta Meghe Institute of Higher Education and Research, Wardha, IND

**Keywords:** masseter muscle, oral carcinoma, salivary gland disorders, strain elastography, temporomandibular disorders

## Abstract

Strain elastography, a non-invasive imaging technique complements traditional diagnostic methods by offering quantitative and qualitative information about soft and hard tissues within the oral cavity. The article aimed to provide an overview of the currently available data on the use of strain elastography in dentistry. To support the review of strain elastography applications in dentistry, a wide range of articles was searched using both online and offline databases. Inclusion and exclusion criteria were defined according to the Population, Intervention, Comparison, Outcomes, and Study Design (PICOS) approach. The results show that 12 of the 107 papers found to be eligible for inclusion in a qualitative examination of the use of strain elastography in dentistry satisfied the PICOS criteria. Elastography is a promising tool for diagnosing various dental diseased conditions, but sufficient evidence is not available. More studies on a larger population should be performed to determine its accuracy in diagnosis.

## Introduction and background

**Figure 1:**
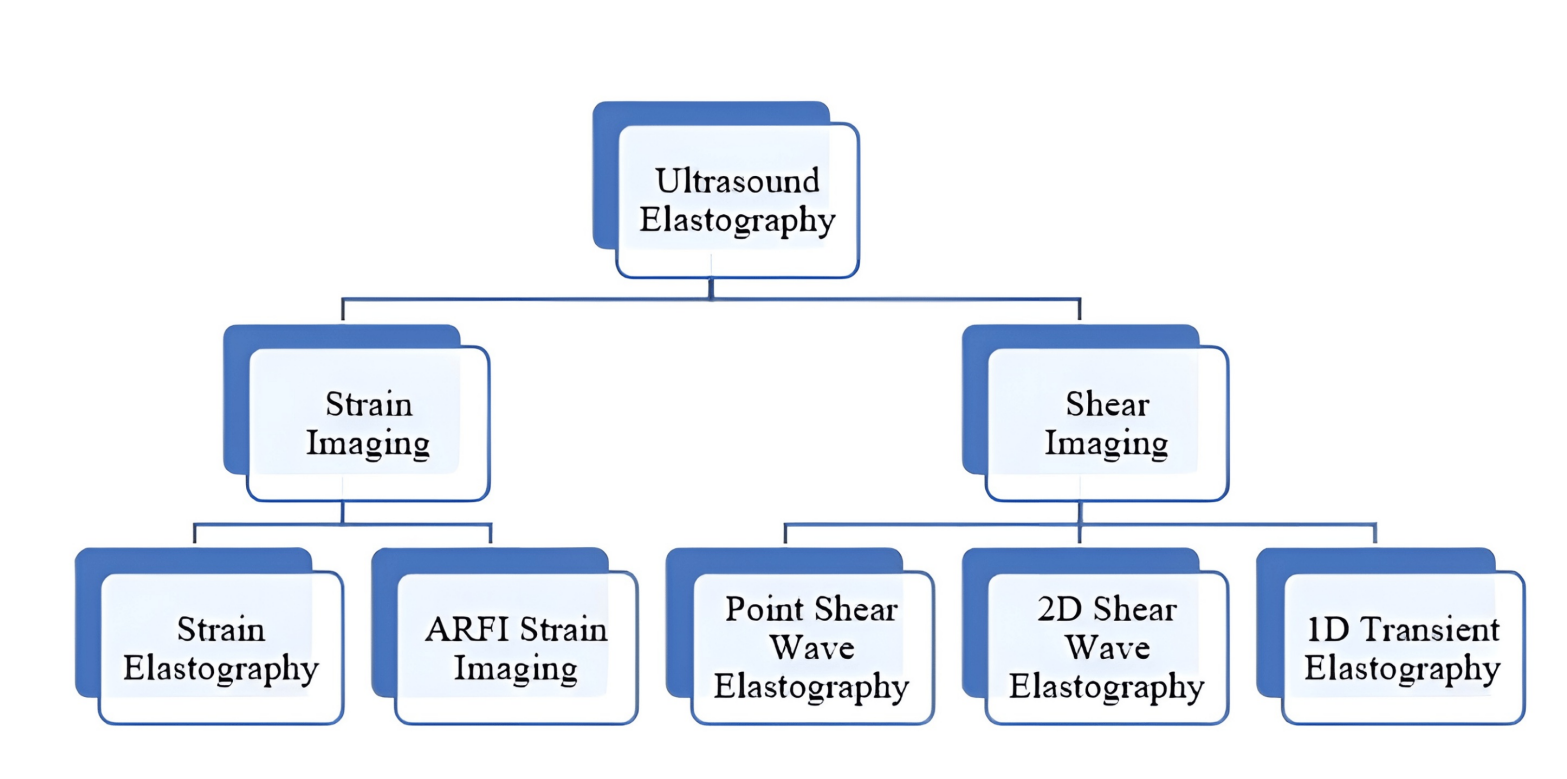
Classification of elastography techniques. ARFI: acoustic radiation force impulse

A non-invasive technique for assessing strain and the distribution of elastic modulus in soft tissues is called elastography (Ey). Ey, in simple terms, is the study of tissue stiffness and how deviations from normal levels are related to diseases of the examined tissues or organs [1]. Ultrasound Ey techniques fall into the following two categories: quasi-static or strain-based, and dynamic or shear wave-based, depending on the type of external mechanical stimuli as depicted in Figure 1.

By applying external tissue pressure, strain elastography (SEy) quantifies the stiffness of the tissue. Strain is the term used to describe the deformation caused by applied pressure on tissue dimensions. Lesions that are more rigid show less deformation, less strain, and a higher Young's modulus. The strain ratio can be calculated by dividing the strain in one tissue region by the strain in the reference tissue region [2]. The underlying concept is that radio frequency (RF) signals must first be obtained before a transducer can apply force to slightly compress the tissue. The pre- and post-compression picture dataset’s RF signals are examined and correlated with one another. The rate of change of tissue displacement and the depth or distance from the transducer are compared in the following step. As a result, a soft lesion moves more than a hard lesion does because soft lesions exhibit both displacement and deformation while hard lesions move as a single unit without changing [3].

SEy has become a highly promising non-invasive diagnostic technique in the medical field, offering a broad range of applications. It works especially well for diagnosing and treating liver and kidney fibrosis, characterizing thyroid nodules and prostate cancer lesions, and identifying benign from malignant lymph nodes in patients with breast cancer [4]. Overall, SEy's ability to deliver real-time, detailed tissue information makes it an essential tool in the diagnostic arsenal across various medical specialties.

Drawing from its applications in the medical field, SEy can significantly benefit dentistry in several key ways. It helps to measure masseter muscle elasticity in periodontitis and temporomandibular disorders. It can also assist in distinguishing between benign and malignant oral lesions, such as tumors or cysts, improving diagnostic accuracy and treatment planning.

This study aimed to comprehensively evaluate the application of SEy in dentistry, highlighting its effectiveness in diagnosing, monitoring, and managing various dental conditions. This review seeks to synthesize current research, identify the benefits and limitations of SEy in dental practice, and provide recommendations for future research and clinical implementation.

## Review

Evidence acquisition

From March 2022 to May 2024, the databases PubMed, Ovid MEDLINE, Web of Science, Scopus, Google Scholar, and ClinicalTrials.gov were searched for pertinent literature. There were no geographical or temporal limitations; only English-language publications were taken into consideration. Keywords including ultrasonic Ey, strain Ey, Sono-Ey, temporomandibular disorder (TMD), masseter, and Ey in dentistry, along with their combinations and variants, were used in electronic search tactics. The inclusion and exclusion criteria were created using the Population, Intervention, Comparison, Outcomes, and Study Design (PICOS) methodology (Table 1). The literature search was carried out by three reviewers. After validating data and abstracts, two reviewers suggested studies for qualitative analysis. The author and the year of publication, the features of the population being examined, the apparatus, and the elasticity values of the tissues under investigation were extracted from a subset of the publications (Table 2).

**Table 1:** Inclusion and exclusion criteria. PICOS: Population, Intervention, Comparison, Outcomes, and Study Design; SEy: strain elastography; RCT: randomized controlled trial; OSMF: oral submucous fibrosis

PICOS	Inclusion criteria	Exclusion criteria
Population	Healthy patients, phantoms, humans and animals, patients suffering from TMD, OSMF, tumors of the salivary gland, palate, and carcinoma	Patients suffering from other than dental-associated pathologies
Intervention	SEy	Shear-wave Ey
Comparison	None or any	-
Outcome	Elasticity or hardness of the affected muscle, tissues, and salivary glands	-
Study	RCTs, cohort studies, systematic reviews, experimental models, animal models, and case reports	Review papers, letters, commentaries, and articles not in English

**Table 2:** Summarization of the studies included in the systematic review. OSMF: oral submucous fibrosis; SR: strain ratio; VAS: visual analog scale; TSCC: tongue squamous cell carcinoma; OS: overall; DCLNM: delayed cervical lymph node metastasis; CTG: connective tissue grafting; TMD: temporomandibular disorder; OA: osteoarthritis; TMJ: temporomandibular joint; BIC: bone-implant contact ratio; BII: bone-implant interface

Studies	Study population	Intervention	Outcome	Conclusion
Ariji et al. (2010) [5]	15 TMD patients (10 with unilateral and five with bilateral muscle pain) used an oral rehabilitation robot to massage their bilateral masseter and temporal muscles alternately.	Sonography was used to assess the masseter's thickness and the presence of anechoic regions both before and after therapy.	20 muscles (66.7%) had anechoic areas before therapy, while 17 muscles (85.0%) had smaller anechoic areas following treatment. The VAS ratings for post-treatment muscular soreness and massage impression were substantially correlated with the pre-treatment thickness.	The treatment effectiveness with regard to muscular discomfort may be correlated with the thickness of the masseter and the presence of anechoic areas.
Damian et al. (2016) [6]	1 case report of unilateral temporal myositis heralding polymyositis.	Determine how Ey helps to distinguish between hypertrophy and inflammation in the muscle.	Moderate elasticity with striated appearance, but no values were provided.	Ey could also be also a useful tool in the management of myositis, but further studies are awaited.
Reichel et al. (2018) [7]	Affected and contralateral healthy salivary glands of 129 Caucasian patients (64 males and 65 females with a mean age of 44.8 years) with sialolithiasis were included.	Ey is used in patients with sialolithiasis for both the first assessment and follow-up during therapy.	Significantly lower values were seen in sialolithiasis-affected submandibular or parotid glands compared to healthy contralateral glands in the same subjects. In comparison to the corresponding healthy contralateral glands, the values of submandibular glands with a single calculus measuring more than 5 mm in diameter or with numerous calculi, and parotid glands with calculi displaying dimensions of more than 5 mm, were substantially higher.	For the objective evaluation of the severity and course of the disease in patients with sialolithiasis, ultrasound Ey offers a simple, fast, and accurate diagnostic technique that can be easily incorporated into already-existing ultrasound procedures.
Elbeblawy et al. (2020) [8]	21 healthy adult volunteers and 21 patients with significant salivary gland inflammatory chronic diseases.	Assess chronic inflammatory conditions of major salivary glands.	Compared to the control group, cases had a median strain ratio of the parotid and submandibular glands that was statistically substantially greater. The strain ratio displayed 97.6% diagnostic accuracy with a sensitivity of 95.2% and specificity of 100% at a cut-off value of 1.13.	The major salivary glands can be diagnosed with chronic inflammatory diseases using Ey.
Taşdemir et al. (2020) [9]	At the beginning of the trial, 124 patients (63 with gingivitis and 61 with chronic periodontitis) were enrolled; however, only 84 patients were confirmed as final participants.	Examine the differences in the masseter muscle's flexibility and thickness between periodontitis and gingivitis patients.	Both during contraction and at rest, the masseter was noticeably thicker in the gingivitis group. Furthermore, the masseter thickness in the gingivitis group was considerably larger during contraction than it was at rest.	The masseter's morphology can be assessed using various Ey modalities.
Mukul et al. (2019) [10]	27 clinically diagnosed and staged participants with OSMF.	Determine how ultrasonic Ey might be used to measure the clinical presentation objectively concerning the severity of the clinical condition in OSMF.	The results showed an extremely significant connection of 0.007 between the clinical and elastographic grading systems. The diagnostic method's sensitivity and specificity were 20% and 90.9%, respectively.	It appears that there are potential benefits to using ultrasound Ey as a diagnostic tool instead of the arbitrary clinical approach for OSMF diagnosis and staging.
Heriveaux et al. (2019) [11]	Coin-shaped implants were crushed using 18 trabecular bovine bone samples.	Utilizing the quantitative ultrasonography technique (QUS), ascertain whether implant stability is susceptible to the loading circumstances at the BII.	During the elastic compression of the trabecular bone samples and the collapse of the trabecular network, there was a considerable decrease in the reflection coefficient of the BII as a function of the compressive stress. The average slope of this decline was -4.82 GPa⁻¹. Changes in the characteristics of the bone material and compression of trabecular bone onto the implant may account for the observed outcomes in terms of an increase in the BIC.	The study quantifies the influence of compressive stresses applied to the BII on its ultrasonic response.
Ogura et al. (2020) [12]	2 clinical cases of palatal tumors.	Intraoral ultrasonography, CT, and MRI were combined with strain Ey to evaluate palatal tumors for both cases.	The strain in the first case - which was identified as a myoepithelioma - was found to be 0.000% (strain of normal tissue=0.556%). The second case had a strain of 0.000% (strain of normal tissue=1.077%), and it was diagnosed as an adenoid cystic carcinoma.	Assessing palatal lesions can be aided by intraoral strain Ey.
Olchowy et al. (2020) [13]	16 of 142 studies identified were analyzed.	Ey is used to evaluate the masseter muscle in both healthy people and those who have problems related to the masseter muscle.	Elasticity values in healthy individuals demonstrated a relationship between the left and right masseter muscle sides, but not in TMD sufferers. Elasticity values rose with TMD and were associated with a higher level of symptom severity. Phantom investigations demonstrated Ey's high degree of dependability.	There is not enough data to conclude that Ey is a useful method for evaluating the elasticity of the masseter muscle. To ascertain whether Ey is a reliable method of characterizing masticatory muscle abnormalities, bigger group studies are required.
Arıkan et al. (2023) [14]	Comprises 40 healthy people and 40 patients who had joint pain and were later identified with TMJ OA using diagnostic cone-beam computed tomography.	In patients with TMJ OA, assess the masseter muscle's hardness and thickness.	At maximum biting, the masseter muscle's mean thickness was 1.28 cm in patients with OA and 1.36 cm in healthy people. OA patients had a mean masseter elasticity index ratio of 4.51 at maximum bite, compared to 3.16 in healthy controls.	For patients with TMJ OA, ultrasonography may be a useful diagnostic technique because it does not require ionizing radiation.
Shibata et al. (2023) [15]	55 patients with muscle-layer invasion and pathologic stage pT1 or T2 TSCC were included in the retrospective analysis.	Assess stiffness as a TSCC prognostic factor.	For the prognosis of DCLNM and OS, the SR cutoff values that maximized the significance of the difference were 7.10 and 7.49, respectively. SR, age, pT stage, depth of invasion, and perineural invasion were found to be significant risk factors for DCLNM in univariate analysis, while SR, sex, and DCLNM were found to be associated with OS. SR was a non-significant but significant risk factor for OS (HR=8.774; p=0.073) and a substantial risk factor for DCLNM (hazard ratio=3.102; p=0.021) in multivariate analysis.	For patients with muscle-layer invasion and pT1/T2 TSCC, tongue stiffness is a predictive feature.
Tavelli et al. (2024) [16]	Includes 28 individuals and 1 clinically competent dental implant showing soft tissue dehiscence.	Examine the changes in ultrasonographic tissue elasticity following CTG for peri-implant soft tissue augmentation at the locations of teeth and implants.	The strain ratio, or SR1, was 0.20±0.08 and 0.30±0.14 at implant sites and in the normal dentition, respectively (p=0.002). This suggests that the coronal region of the soft tissue surrounding teeth is typically more elastic than the surrounding soft tissue surrounding dental implants. During 12 months, the midfacial coronal part of the soft tissue showed increased stiffness due to soft tissue augmentation with CTG (p<0.001 for SR1, SR2, and SR3).	Tissue elasticity and related alterations during soft tissue augmentation are recorded and quantified by ultrasound strain Ey. A modality of importance to illustrate the dynamic and synergistic influence of the soft tissue phenotypic components is ultrasonography tissue elasticity.

Results

Evidence Synthesis

**Figure 2:**
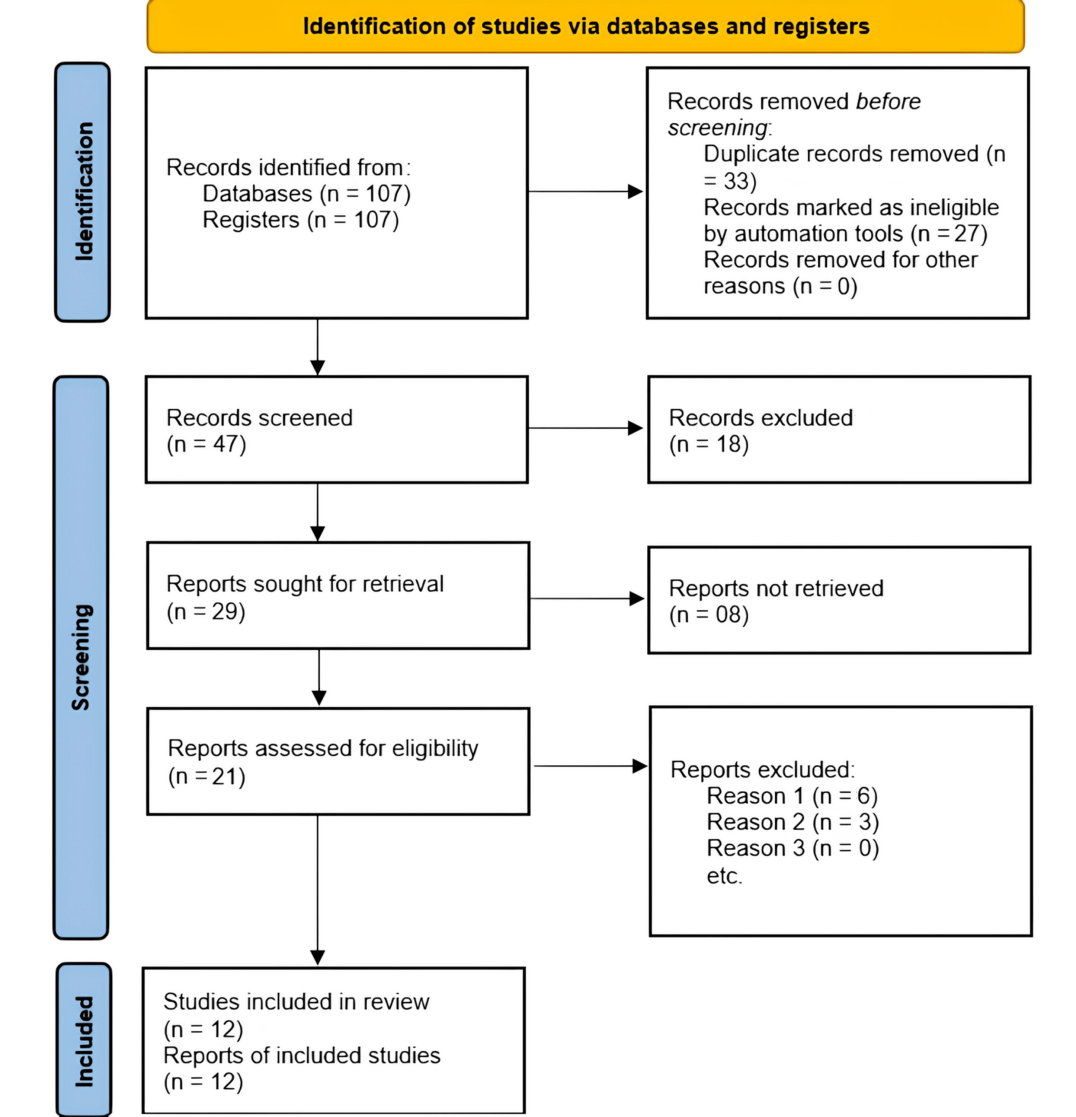
The flow diagram depicting the selection of article.

Twelve of the 107 papers that were found to be eligible for inclusion in a qualitative examination of the use of SEy in dentistry satisfied the PICOS criteria. The list of the primary articles had been imported into EndNote software (Philadelphia, PA: Clarivate Analytics), and duplicates were eliminated. Ten publications were considered for the evaluation of the strain Ey values. A flow diagram of the selection process is given in Figure 2. Table 2 shows the data matrix.

Discussion

Masseter Muscle

A variety of treatments have been proposed for fascial pain. Physical therapy relieves musculoskeletal discomfort and restores normal function by reducing inflammation, decreasing and coordinating muscular activity, and encouraging tissue repair and regeneration [17]. Massage is a strong mechanical assistance that activates the pain gating mechanism. It can alleviate a natural discomfort, promote a higher release of opiates, and achieve more substantial pain relief [18].

The muscle elasticity index is a measure of how a muscle responds to stress or deformation, specifically its ability to recover to its normal shape after stretching. The exact normal range for the masseter muscle varies based on age, gender, and health status, but a healthy, non-contracted masseter muscle should have some flexibility that allows it to stretch and return to its resting position quickly. Sonographic characteristics could be used to determine the therapeutic efficacy of massage therapy [5].

Periodontitis is widely recognized to produce mobility and loss of oral functions; thus, it may impact masticatory ability, which can be assessed using bite force and masseter muscle thickness [19]. According to the studies, various Ey modalities can be utilized to assess the masseter's morphology. Ey is a promising method for assessing masseter muscle elasticity to enhance TMD diagnosis and monitor treatment outcomes; nevertheless, the data are not abundant [13].

Temporal Muscle

Temporal myositis is an uncommon inflammatory illness of the temporal muscle characterized by local swelling and pain. Ultrasonography is a reliable approach for assessing temporal muscle disease [20]. In the presence of temporal pain and swelling, ultrasonography may aid in diagnosing, determining the extent of the lesion, and directing muscle biopsy. It also contributes to the elimination of alternative etiologies, such as vasculitis, malignancies, and infections, as well as potentially monitoring disease development [6].

Disorders of the Salivary Gland

The most common condition affecting the salivary glands is inflammation, which can arise from a variety of circumstances. It manifests in autoimmune diseases of the salivary glands, including Sjogren's syndrome, sialolithiasis, and sialadenosis. The appearance could be acute or chronic, depending on the underlying etiology [21]. Patients with chronic inflammatory illnesses experience increased stiffness and, consequently, higher elasticity values due to the progressive acinar degradation brought on by the chronic inflammatory process, which is also accompanied by a lymphocytic infiltrate, fibrous replacement, and sialectasis. Acoustic radiation force impulse Ey was used in the first assessment and follow-up of patients with sialolithiasis. It offers a quick, simple, and accurate diagnostic method for determining the structural tissue condition of salivary glands impacted by sialolithiasis [22].

Oral Submucous Fibrosis (OSMF)

OSMF is a recognized "potentially malignant" condition with an incidence rate of 0.2-1.2% in India. The biopsy is an intrusive technique that results in surgical trauma and tissue scarring, which adds to the severity and progression of OSMF. Ey is a non-invasive, simple, and exact technique for determining the degree of fibrosis by measuring tissue stiffness. It can be the most useful instrument when mouth opening is very limited or absent [10].

Oral Carcinoma

Initial SEy findings have been generally favorable; it has been found to produce reasonably accurate results for malignancies in a variety of places across the body, owing to the fact that malignancies are stiffer than benign diseases and normal tissues [23]. Sonography of the tongue can indicate the nature of a tongue lesion, such as its border, size, location, depth, presence or absence of a capsule, and interior structure, including vascularity [24]. Furthermore, ultrasonographic findings closely resemble histology findings [25]. According to Lyshchik et al., sono-elastography (sonoEy) was highly accurate in distinguishing between cervical lymph nodes that were benign and those that were metastatic when it came to patients who might have thyroid or hypopharyngeal cancer [26]. Ishii et al. indicated that intraoral ultrasonography of palatal tumors could be used to determine the localization and condition [27]. The firm connection between ultrasonographic data and histology results demonstrates the reliability of SEy. In dentistry, SEy could be a useful addition to established diagnostic methods, perhaps lowering the need for invasive treatments by providing a non-invasive way to analyze tissue features and pathology.

Peri-Implant Tissues

The soft tissue phenotype has been demonstrated to play an important function in teeth and dental implants. The impact of the soft tissue phenotype on periodontal/peri-implant health, esthetics, and soft tissue margin stability has primarily been studied by examining the implications of its components, namely keratinized tissue width and mucosal thickness. It has been hypothesized that one of the major properties of the periodontal/peri-implant soft tissue is its potential to form a "seal" around the tooth/implant, which could improve health, esthetics, and soft tissue stability, regardless of the levels of the underlying buccal bone [28]. To better understand the interaction between the various elements of the soft tissue phenotype and to further investigate the dynamic behavior of the periodontal/peri-implant soft tissue when compression forces are applied, ultrasonographic imaging can be used around teeth and dental implants. Similarly, given that sites diagnosed with peri-implantitis are frequently characterized by a movable - and therefore more elastic than stiff - peri-implant soft tissue at the midfacial aspect due to the inflammatory infiltrate, it is also possible to assume that tissue elasticity may also reflect the health condition of dental implants [29].

The development of the biomechanical characteristics of the bone-implant interface (BII) directly affects the outcome of implant surgery [30]. Several biomechanical methods have been developed for the evaluation of implant stability. Resonance frequency analysis (RFA), the most widely utilized biomechanical technique, involves determining the implant's tiny rod's first bending resonance frequency [31]. It's interesting to note that the quantitative ultrasonography technique (QUS) has become a viable technique for obtaining BII data. Recent research has demonstrated that the QUS device's sensitivity and reproducibility outperformed the outcomes of RFA in vitro and in vivo by a large margin [32,33]. Tissue stiffness changes during post-operative tissue recovery, especially after connective tissue grafting. Sites affected by peri-implantitis often show reduced stiffness and increased tissue mobility. SEy works well for detecting alterations over time and assessing the peri-implant tissues' elasticity. This could facilitate early peri-implantitis diagnosis and enable timely treatment.

## Conclusions

SEy offers valuable insights into the mechanical properties of dental tissues, making it a versatile tool for diagnosing, treating, and monitoring various dental conditions. Its applications range from early detection of oral cancers to evaluating the success of dental implants and guiding surgical procedures. As technology advances, SEy will likely become an increasingly important component of dental diagnostics and treatment planning.

Despite its potential, the broad application of SEy in dentistry necessitates additional research and standardization. Clinical investigations are required to provide uniform methods, precision, and dependability in a variety of dental applications. Furthermore, integration with other imaging modalities and technological advancements could increase its usefulness. As more data becomes available, strain elastography has the potential to transform dental diagnostics, making it an indispensable tool for enhancing patient treatment and outcomes in the future.
